# Alexithymia, Phosphorus Levels, and Sleep Disorders in Patients on Hemodialysis

**DOI:** 10.3390/jcm11113218

**Published:** 2022-06-05

**Authors:** Đorđe Pojatić, Dajana Nikić, Ivana Tolj, Davorin Pezerović, Andrijana Šantić, Dunja Degmečić

**Affiliations:** 1 Faculty of Medicine Osijek, Josip Juraj Strossmayer University of Osijek, 31 000 Osijek, Croatia; djolepojatic@gmail.com (Đ.P.); ivanatolj5@gmail.com (I.T.); davorin.pezerovic@obvk.hr (D.P.); andrijana.miskovic1@gmail.com (A.Š.); 2 Faculty of Dental Medicine and Health Osijek, Josip Juraj Strossmayer University of Osijek, 31 000 Osijek, Croatia; 3 Department of Internal Medicine, General County Hospital Vinkovci, 32 100 Vinkovci, Croatia; 4 Department of Plant Sciences, Faculty of Biosciences, Norwegian University of Life Sciences, P.O. Box 5003, N-1432 As, Norway; blagojevicd90@gmail.com; 5 Department of Internal Medicine, University Hospital Osijek, 31 000 Osijek, Croatia; 6 Department of Psychiatry, University Hospital Osijek, 31 000 Osijek, Croatia

**Keywords:** alexithymia, daytime sleepiness, depression, inflammation, phosphorus levels

## Abstract

Alexithymia, or the inability to distinguish between bodily feelings and emotions, has been linked to poor sleep quality in some studies. Rare studies examined the associations between electrolyte phosphorus in patients on hemodialysis and their sleep quality, daytime sleepiness, and alexithymia with inflammatory factors. Hemodialysis is a treatment method for terminal renal patients that involves the diffusion of unwanted metabolic products through the dialyzer membrane. Our study aimed to examine whether there was a difference in phosphorus levels, inflammatory factors, and daytime sleepiness according to the hemodialysis patients’ levels of alexithymia. The study involved 170 HD patients that had been treated with chronic dialysis for more than three months. Prior to the hemodialysis procedure, laboratory findings were sampled. Respondents completed the Pittsburgh Sleep Quality Index, the Toronto Alexithymia Scale 26, and the Epworth Sleepiness Scale, and were questioned about depression. The results showed that alexithymic HD patients exhibited significantly higher leukocyte counts, lower predialysis phosphorus values, and more pronounced daily sleepiness than the alexithymia-free group (Mann–Whitney U test, *p* = 0.02, *p* = 0.005, and *p* < 0.001, respectively). We concluded that alexithymia was an independent predictor of high daytime sleepiness in HD patients (OR = 1.05, 95% CI 1.02 to 1.09).

## 1. Introduction

Chronic kidney disease (CKD) is defined as a state of decreased glomerular function with creatinine clearance lower than 60 mL/min/1.73 m^2^ and a duration of more than three months [1]. CKD is classified based on the degree of glomerular filtration; consequently, five stages of CKD are distinguished, with the fifth stage, known as the end stage, referring to creatinine clearance of less than 15 mL/min [2]. Chronic hemodialysis treatment is initiated in stage 5 CKD, when the glomerular filtration rate is between 7 and 9 mL/min, taking into account other clinical indicators, such as the etiology of the underlying disease, presence of hypervolemia, hyperkalemia, metabolic acidosis, and patient’s comorbidities [3].

Individuals on a chronic hemodialysis treatment program face a variety of problems on a daily basis as a result of their unique lifestyle, which requires them to limit their food and fluid intake, regularly take dozens of medications, and limit their time spent on professional and family development [3]. In addition to physical restrictions, factors such as secondary anemia and secondary hyperparathyroidism considerably diminish the quality of life of HD patients [4,5]. A high prevalence of sleep quality disorders (41–83%) stands out, with it being several times higher than in the general population and having a negative impact on HD patients’ survival [6,7]. Sleep quality is a generally accepted concept of sleep evaluation that includes several of its key components, specifically, general sleep quality, sleep latency, duration and effectiveness of sleep, sleep disturbance, medication use, and daytime sleepiness [8]. One study on HD patients with severe secondary hyperparathyroidism found a negative correlation between such a comprehensive sleep evaluation and alexithymia, a term denoting difficulty in identifying and describing emotions to others and externally oriented thinking [9]. Aside from the 2010 study by De Santo et al., there are no findings on the relationship between alexithymia and sleep quality in HD patients, and as far as we are aware, there are no studies on the relationship between alexithymia and the concept of daytime sleepiness [9]. Additionally, daytime sleepiness is significantly higher in HD patients than in the general population, and its relationship with alexithymia has not been studied [10]. Given the significantly higher prevalence of alexithymia in the HD patient population, it is reasonable to expect that its association with daytime sleepiness would be even stronger and clinically more significant than in the general population and should thus be investigated.

Previous studies related to HD patients showed a highly increased alexithymia prevalence, ranging from 13% to 80%, with the highest alexithymia prevalence noted in the studies examining patients with severe comorbidities, such as secondary hyperparathyroidism and diabetes [9,11]. Furthermore, a common link between the above-mentioned comorbidities and end-stage CKD is the high level of oxidative stress, which is a predisposing factor for the development of systemic inflammation [12].

On the other hand, in contrast to alexithymia, the correlation between systemic inflammation and sleep quality in HD patients was thoroughly investigated. Some of the relevant studies found a negative correlation between CRP and sleep quality in HD patients [13]. However, other studies demonstrated contradicting results; therefore, based on the above, it is necessary to examine the relationship between systemic inflammation, alexithymia, and sleep quality in a large enough sample of HD patients. There is a possibility that systemic inflammation, such as that seen in depression due to atrophy of specific areas of the brain parenchyma, is associated with alexithymia and poor sleep quality [14].

According to the DSM-5 classification, depressive disorder is defined as the presence of a depressed mood or loss of interest in everyday activities persisting for at least two weeks. The prevalence of depressive disorder in HD patients compared with the general population and other patient samples is significantly higher; 10% to 44.8% of HD patients have some form of depressive disorder [13]. The particularity of depressive disorder in HD patients is that the symptoms of depression frequently overlap with the symptoms of uremia, which is why it is necessary to use depression assessment instruments that exclude this fact when assessing symptoms of depression. In previous studies, depression was defined as a factor independent of alexithymia in the population of HD patients; it was linked to high levels of inflammation and sleep quality disorders in HD patients, while evidence of the connection between and influence of depression on daytime sleepiness in HD patients was not found [15,16].

Daytime sleepiness is a factor that is closely related to sleep disorders in HD patients, which can have a substantial impact on their safety and quality of life and is the least investigated compared to other sleep disorders in HD patients [17]. According to the international classification of sleep disorders, excessive daytime sleepiness is the inability to stay awake or alert during the day, resulting in unintended sleeping that occurs for at least three months [18]. Compared with the general population, excessive daytime sleepiness in the HD patient population is significantly higher. Daytime sleepiness is a factor that can severely limit HD patients’ ability to perform routine daily activities, including self-care and household tasks, and previous research failed to identify the factors that are closely associated with it in the HD patient population [10]. Previous studies did not investigate the relationship between daytime sleepiness and interdialytic weight gain in HD patients. Such weight gain could be the cause of altered daytime sleepiness in the HD patient population, as it should inevitably be associated with blood pressure values, which, when lowered, cause sleepiness in the general population.

Given the high prevalence of alexithymia in individuals with uncontrolled secondary hyperparathyroidism, there is some debate about its association with electrolytes, such as phosphorus [9]. One cross-sectional study found a negative correlation between alexithymia and phosphorus values prior to dialysis, but the study was conducted on a small sample and was not randomized [19,20]. Given the negative correlation between phosphorus, cardiovascular morbidity, and HD patients’ survival, this finding definitely requires additional research on a large enough sample of HD patients [21]. The knowledge gathered in the general population concerning alexithymic individuals’ higher propensity for an unhealthy diet, smoking, and sedentary lifestyle leads to assumptions about the influence of alexithymic personality traits on the health status of HD patients [22]. If an HD patient has difficulty distinguishing between emotions and somatic sensations as a result of alexithymic personality traits, due to their proneness to uncontrolled fluid and food intake, they are more likely to have higher electrolyte values and interdialytic weight gain that endangers their cardiovascular system.

Thus, the main goal of this study was to investigate sleep quality, daytime sleepiness, depression, and laboratory variabilities (levels of serum phosphorus, leukocytes, and CRP) and interdialytic weight gain in HD patients on a chronic hemodialysis treatment program with respect to the measured levels of alexithymia and to examine which factors most affected the daytime sleepiness of HD patients. The specific hypotheses of our study are that alexithymic HD patients suffer more from daytime sleepiness and depression; have lower sleep quality; increased levels of serum phosphorus, leukocytes, CRP, and interdialytic weight gain than non-alexithymic HD patients; and that alexithymia is an independent predictor of excessive daytime sleepiness in HD patients.

## 2. Materials and Methods

This was a multicentric, cross-sectional study that was approved by each institution’s Ethics Committee after a careful assessment of all the methods used. The research was approved by the Ethics Committees of the General County Hospital Vinkovci (under the code 01-9206/3/19), Clinical Hospital Centre Osijek (under the code R2-1060/2020), and Health Center Županja (under the code 381-12-19-01-1).

### 2.1. Participants

The research was conducted among HD patients in the hemodialysis treatment program at the Department for Hemodialysis of the General County Hospital Vinkovci, Department for Nephrology of the Clinical Hospital Centre Osijek, and Dialysis Unit of the Health Center Županja.

The sampling of participants was performed by random selection of slips with dates of the patients’ first hemodialysis from a “drum”. Each participant signed informed consent for participation in the survey after a clear explanation of every detail of it. Questionnaires and blood samples taken prior to the hemodialysis treatment were coded with a unique 8-digit number (date of the HD patient’s first dialysis). The unique eight-digit code also enabled anonymous access to the HD patients’ medical documentation, marked with said codes according to medical protocol. Patients were given questionnaires to assess alexithymia and sleep disorders, while a psychiatrist assessed for depression symptoms. The survey was conducted in February 2020 and lasted for twelve months.

### 2.2. Selection Criteria for Participants in the Study

The study included HD patients of both sexes, over 18 years of age, receiving hemodialysis and hemodiafiltration at least twice a week for more than three months, whose participation was voluntary.

**Figure 1:**
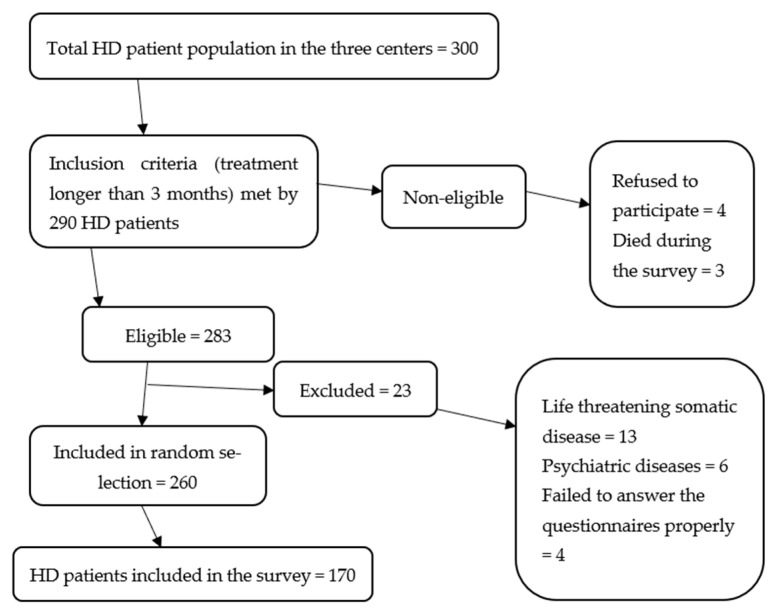
Flowchart of patient inclusion.

Excluded from the study were HD patients who did not complete the questionnaires properly or were diagnosed with psychiatric disorders or severe somatic conditions (cardiovascular diseases, such as myocardial infarction and heart failure, liver cirrhosis, neoplasms, limb amputations) within three months before the survey (Figure 1).

### 2.3. Evaluated Variables and Methods

Inspection of medical records revealed the total duration of hemodialysis treatment, the shift in which an HD patient was dialyzed, the duration of individual hemodialysis treatments, and the average systolic and diastolic pressure at the end of the treatment for the last two/three hemodialysis treatments [23]. HD patients’ interdialytic mass yield was calculated upon reviewing the medical records, according to the following formula: patient’s body weight at the end of the previous dialysis—the patient’s body weight at the time of the next dialysis/(HD patient’s dry body weight + number of days between two dialysis treatments for two/three hemodialysis treatments during the week in which the survey was conducted), and the arithmetic mean of the two or three measured values was used [15]. Laboratory values of blood count, phosphorus, and CRP were measured prior to the hemodialysis treatment [24].

HD patients completed a socio-demographic questionnaire, the Toronto Alexithymia Scale (TAS 26), the Pittsburgh Sleep Quality Index (PSQI), and the Epworth Sleepiness Scale (ESS) for the purpose of the study, while a psychiatrist assessed the intensity of depression symptoms based on clinical observation and using the Hamilton Depression Rating Scale (HDRS) [25].

A sociodemographic questionnaire designed for HD patients consists of items related to general data, such as participants’ age, sex, and education level [16]. To measure the sleep quality, HD patients completed a self-assessment PSQI questionnaire grouped into 7 components that assess subjective sleep quality, sleep latency, sleep duration, habitual sleep efficiency, use of sleep medication, daytime dysfunction, and sleep disturbance in the last month [26]. Each component can be scored from 0 to 3, and the overall component score is 21. The higher the result, the lower the sleep quality. If the participant scores 5 or more points, they are classified as having low sleep quality [27]. To measure daytime sleepiness, we used the ESS, which is a self-assessment questionnaire consisting of eight items, each of which assesses the subjects’ daytime sleepiness in the offered hypothetical situations [26]. Each question can be scored from 0 to 3, where a higher score means a higher proneness to doze off in hypothetical situations [6]. The questions are designed affirmatively and should be graded so that a higher score means higher daytime sleepiness. If the participant scores 10 or more points, they are classified as having increased daytime sleepiness [26]. To assess depression symptoms, the Hamilton Depression Rating Scale (HDRS) was used, which is validated for use in Croatian and has good internal consistency (Cronbach’s α = 0.90). The Scale consists of 17 items that are scored positively on the basis of answers and observation of the participant; it classifies patients based on the degrees of depression symptoms. A psychiatrist assessed depression symptoms using the Scale [25].

TAS 26 was used to measure alexithymia. TAS 26 is a self-assessment scale consisting of 26 items organized in four subscales that measure difficulty identifying feelings, difficulty describing feelings, reduced daydreaming, and externally oriented thinking [28]. Participants respond to questions on a Likert-type scale of 5 points, agreeing or disagreeing on how much a fact from the question represents them (1 = completely agree, 5 = completely disagree). Patients who score between 63 and 72 points are moderately alexithymic, while those who score more than 73 points are alexithymic. Participants who score 62 points or less are not alexithymic [27]. TAS 26 was used because it was validated on a clinical sample of patients in Croatian, unlike other instruments for measuring alexithymia [27].

To detect significant differences between nonparametrically distributed numerical data in two groups of subjects, we calculated the minimum required sample size of 160 HD patients, 113 in the group of HD patients without alexithymia and 47 alexithymic HD patients, assuming a significance level of 0.05, mean effect (Cohen’s d) of 0.5, test power (1–β) of 0.8, and a ratio of alexithymic and HD patients without alexithymia of 0.42 (according to the result of a priori analysis). For the purpose of statistical analysis, HD patients were divided into groups of alexithymic patients, which included HD patients with alexithymia and moderate alexithymia, and non-alexithymic patients, based on their TAS 26 results, into good and bad sleepers based on their PSQI results, and into those with normal and excessive daytime sleepiness based on their ESS results.

### 2.4. Statistical Methods

Categorical data are presented via absolute and relative frequencies. Differences between categorical variables were tested using the x2 test and, where necessary, using Fisher’s exact test. Numerical variables were tested for distribution normality using the Shapiro–Wilk test. Numerical data were described using median and interquartile range limits for distributions that did not follow the normal distribution and using the arithmetic mean and standard deviation in normally distributed data. The means of the numerical variables of interest were rated with a 95% confidence range (95% CI). The Mann–Whitney U test (with the Hodges–Lehmann median difference) was used to test the differences between numerical variables in two independent groups of subjects, while the independent samples *t*-test (Levene’s test for equality of variances) was used to test the differences between normally distributed numerical data of the two groups. The correlations between the numerical variables were measured using Spearman’s rank correlation coefficient ρ (rho). The effect of alexithymia on serum phosphorus levels was examined using linear regression. Logistic regression was used to assess the impact of several factors on the probability of daytime sleepiness to control for confounding, such as depression, hemoglobin levels, average systolic and diastolic blood pressure, and interdialytic weight gain (bivariate and multivariate logistic regression) [29]. All *p*-values are presented as two-sided *p*-values. The level of significance was set at α = 0.05. The SPSS software package was used for the data analysis (IBM Corp. Released 2015. IBM SPSS Statistics for Windows, Version 23.0. Armonk, NY, USA, IBM Corp.).

## 3. Results

### 3.1. Basic Characteristics of the Participants

The study was conducted on 170 participants, 99 (58.2%) of whom were men and 69 (40.6%) were women. The median age of the participants was 67 (interquartile range of 58 to 74), with a 20 to 87 range. A total of 77 (45.3%) participants had a secondary school qualification and 53 (31.2%) of them had a primary school qualification. The first and second dialysis shifts were the most common, with only 15 (8.8%) participants being dialyzed in the third shift. A total of 145 (85.3%) participants underwent three dialyses a week, while two (1.2%) participants underwent four dialyses a week. A total of 94 (55.3%) participants had a fistula as the vascular access to the bloodstream. The median duration of dialysis was 2 years, ranging from 90 days to 35 years.

### 3.2. Alexithymia (TAS 26 Scale)

Alexithymia was measured with the TAS 26 questionnaire. The median of the total TAS-26 scale was 68 (interquartile range from 62 to 77), ranging from 27 to 114 (Table 1). Given the values of the TAS-26 scale, 111 (65.3%) participants had alexithymia or interalexithymia (63 points or more); for the purposes of statistical analysis, they are grouped together as HD patients with alexithymia.

There were no significant differences in the ages of HD patients based on levels of alexithymia (difference −2; 95% confidence interval −7 to 2; Mann–Whitney U test, *p* = 0.318) between the groups (Table 2).

There were no significant differences in the levels of alexithymia of HD patients based on their sex (difference 1.33; 95% confidence interval −3.59 to 6.26; independent *t*-test, *p* = 0.594), (Table 3).

### 3.3. Sleep Quality (PSQI Questionnaire)

Sleep quality was assessed using the Pittsburgh Sleep Quality Self-Assessment Questionnaire. The median of the total sleep scale was 6, ranging from 0 to 26 (Table 4).

Given the values of the total sleep scale, 117 (68.8%) participants were poor sleepers (scored 5 or higher).

### 3.4. Epworth’s Daytime Sleepiness Scale

The Epworth Daily Sleepiness Scale (ESS) was used to measure daytime sleepiness. The median of daily sleepiness was 6 (interquartile range from 3 to 10), ranging from 0 to 24. According to the scale values, 35 (20.9%) participants suffered from excessive daytime sleepiness (11 points or more) (Table 5).

### 3.5. Levels of Depression (Hamilton Depression Rating Scale)

Using the Hamilton Scale, the psychiatrist assessed the presence and intensity of depression symptoms. The median of the Scale was 8 (interquartile range between 4 and 13), ranging between 8 as the lowest and 29 as the highest. According to the Scale, 60 (35.3%) participants were not depressed, while 63 (37%) participants had some form of depression. Of these participants, 34 (20%) had mild depression, while severe or very severe depression was present in 14 (8.2%) cases.

### 3.6. Correlation of Interdialytic Weight Gain, Blood Pressure, and Biochemical Indicators with Daytime Sleepiness

We evaluated the correlation of interdialytic weight gain with the daytime sleepiness scale using Spearman’s correlation coefficient. We perceived a significant, negative, and weak correlation of interdialytic weight gain with the Epworth sleepiness scale (rho = −0.205; *p* = 0.01) and vice versa. Participants with excessive daytime sleepiness had somewhat lower levels of interdialytic weight gain, with a median of 0.0124 (interquartile range from 0.0099 to 0.0158) compared with participants without excessive daytime sleepiness, with borderline statistical significance (Mann–Whitney U test, *p* = 0.05) (Table 6).

### 3.7. Correlation of Pittsburgh Sleep Quality Index (PSQI) with Biochemical Indicators and Depression

Significantly higher CRP levels (difference 1.6; 95% confidence interval 0.18 to 3.76; Mann–Whitney U test, *p* = 0.02) and depression levels (difference −5; confidence interval −7 to −2; Mann–Whitney U test, *p* = 0.00) were observed in participants who were bad sleepers according to the PSQI compared with good sleepers (Table 7).

There was no significant association between CRP (rho = 0.14; *p* = 0.866) and leukocyte levels (rho = −0.003; *p* = 0.972) regarding latency to sleep, nor was there a significant relationship between CRP (rho = 0.75; *p* = 0.359) and leukocyte levels (rho = −0.28; *p* = 0.729) with the total sleep duration of HD patients.

### 3.8. Correlation of Alexithymia with Factors of Inflammation, Sleep Quality, Daytime Sleepiness, and Depression

There were significantly higher leukocyte levels (difference 0.9, 95% confidence interval 0.2 to 1.6; Mann–Whitney U test, *p* = 0.02), significantly lower pre-HD phosphorus levels (difference −0.27 with 95% confidence interval −0.44 to −0.08; Mann–Whitney U test, *p* = 0.005), higher daytime sleepiness (Epworth Sleepiness Scale), median 8 (interquartile range between 4 and 11; Mann–Whitney U test, *p* < 0.001) and higher levels of depression (difference −3 with 95% confidence interval −6 to −1; Mann–Whitney U test, *p* = 0.01) in the group of patients with alexithymia compared with patients without alexithymia (Table 8).

### 3.9. Influence of Individual Factors (Predictors) on Serum Phosphorus Levels and Daytime Sleepiness (Regression Analysis)

We tested the functional correlation between phosphorus and alexithymia using regression analysis. Alexithymia explained 7.9% (*p* = 0.001) of the pre-HD phosphorus variability (Table 9).

In order to examine which predictive factors affected the probability of excessive daytime sleepiness in HD patients, we used eleven independent variables (indicated in Table 10). The significance of individual predictive factors was presented using bivariate regression analysis.

As a model, we observed factors that were significant (alexithymia and interdialytic weight gain) together with other chosen factors. The model was statistically significant in its entirety (χ^2^ = 12.862, *p* = 0.002), which showed that it could differentiate between participants based on daytime sleepiness levels. The model as a whole explained between 10.5% (according to Cox and Snell) and 16.6% (according to Negelkerke) of the variance in the clinical presentation of daytime sleepiness and correctly classified 81.0% of cases. Only one independent predictive factor gave a statistically significant contribution to the model, which was alexithymia (OR = 1.051) (Table 10).

## 4. Discussion

The prevalence of alexithymia in the sample of HD patients included in our study was 65.3%, which is one of the highest percentages if we compare our results with the results of previous studies. A higher prevalence of alexithymia, approximately 85%, was present only in one study, in which the sample was small and included only 40 HD patients with severe secondary hyperparathyroidism, while the prevalence was almost equal to the one in a sample of HD patients with diabetic nephropathy [9,11]. Although it is known that alexithymia levels in the general population are significantly higher in men and elderly persons, this was not the case in the sample of HD patients since there were no significant differences observed in the levels of alexithymia based on the sex of HD patients, nor was median age significantly different in groups of participants divided based on the presence of alexithymia [30]. It is therefore possible that HD patients undergoing chronic hemodialysis achieve high levels of alexithymia through other mechanisms and that alexithymia acts as a type of immature defense mechanism against physical stressors [31]. In that case, HD patients bear physical pain more easily in a state of decreased insight into their mental condition and vice versa. This explains the high levels of depression in the sample of HD patients with alexithymia since they cognitively processed psychophysical stressors in an inadequate manner, which resulted in a distorted image of reality and led to decreased coping with reality, high levels of frustration, and the development of depression symptoms; this should also be researched in longitudinal studies [31]. Although we excluded patients with recent acute physical and psychiatric conditions with potentially high levels of somatic pain from our sample of HD patients, a downside of our study was the fact that we did not assess the level of pain that may be related to levels of alexithymia. This remains a task for future researchers.

If we discuss our results based on the prevalence of alexithymia in our sample, we note that there was a statistically significant difference in the total leukocyte count. This means that with higher leukocytosis, alexithymia levels were higher. Based on the results of previous studies, patients with alexithymia are constantly in a state of increased sympathetic tone. Since the sympathetic nervous system is connected to lymph nodes through peripheral nerve endings and circulating catecholamines, it thus affects the activation of immune cells and in situations of acute stress, it activates the Th-1 immune response, which may explain the significantly high leukocyte levels present in our sample [32]. Our hypothesis is supported by the fact that the group of patients with alexithymia is significantly more depressed compared to the group without alexithymia; it is, therefore, also likely in a state of heightened exposure to stress. A downside of our study was the fact that we failed to objectivize levels of stress, which must be divided into acute and chronic stress, in our sample of HD patients since certain studies have established a relationship between the type of stress and the modulation of the immune response toward the Th-1 or Th-2 pattern in patients with alexithymia.

When dividing HD patients into good and bad sleepers based on PSQI results, we confirmed our hypothesis regarding higher CRP levels in a sample of HD patients with sleep disorders, which was in accordance with the results of some previous studies [33]. Even though the difference in leukocyte levels was not significant, leukocytosis was somewhat more pronounced in the group of bad sleepers, which was also in accordance with the hypothesis regarding the positive correlation between inflammation and poor sleep quality in HD patients [34]. Leukocytosis in HD patients with poor sleep quality and significantly high CRP levels likewise corresponds to the hypothesis regarding increased sympathetic nervous system activity in HD patients with poor sleep quality since high sympathetic nervous system activity represents a link between poor sleep quality and heightened systemic inflammation, as well as alexithymia [32]. Up to this point, no research has been conducted that simultaneously studied systemic inflammation levels, alexithymia, and sleep quality in HD patients, which is a major deficiency since their relationship could be similar to the relationship between systemic inflammation and depression in the general population [14]. Systemic inflammation can cause disfunction of the negative feedback of cortisol on an adrenocorticotropic hormone, leading to a loss of inhibitory feedback of inflammatory cytokines and CRP, and thus, leading to atrophy of the limbic system of the brain [35,36]. In addition to this, The low levels of vitamin D that are present in HD patients with sleep disorders cannot inhibit the production of proinflammatory cytokines and CRP [37]. Since HD patients are a special group that is more prone to systemic inflammation compared with the general population due to the characteristics of vascular access and long-term exposure to oxidative stress, it is possible that alexithymia in HD patients is a consequence of the negative effects of inflammation on brain tissue [38]. These assumptions are supported by research on general population individuals who had their alexithymia levels assessed after specific organic impairments. Thus, individuals enduring purposeful interruption of the continuity of the commissural fibers of the cerebrum in order to better control epilepsy were shown to have difficulties identifying emotions in other people’s verbal expressions [39]. The effect of systemic inflammation on sleep quality and alexithymia should be examined in prospective studies since this relationship is a potential cause of high prevalence of alexithymia and poor sleep quality in HD patients.

Phosphorus levels, measured immediately prior to hemodialysis, are higher in HD patients without alexithymia compared with HD patients with alexithymia. One study of HD patients found a similar inverse correlation between alexithymia and phosphorus levels prior to hemodialysis treatment [19]. On the other hand, a cross-sectional, monocentric study conducted by Tayaz and associates did not confirm this correlation [20]. Although both studies addressed the importance of alexithymia and phosphorus levels prior to hemodialysis treatment, the differences in the results are explained by the limited number of participants and monocentric study method given that each hemodialysis facility may have different ways to prevent and treat hyperphosphatemia. In contrast, our study, which was multicentric and was conducted on a three times larger sample of patients, undeniably confirmed a negative correlation between phosphatemia and alexithymia. Alexithymia accounted for nearly 7.9% of the variability of pre-dialysis phosphorus levels; the coefficient was negative. The relationship between alexithymia traits and low phosphorus levels was unexpected for us and was likely under the influence of other, confounding factors that were not controlled for in our study. In HD patients, phosphorus is absorbed in the small intestine, which is more pronounced with the consumption of raw food that is rich in proteins and phosphates and is a factor that affects the progression of mineral and bone disorders in CKD since it induces PTH excretion in addition to hypocalcemia [40]. Since phosphatemia values immediately prior to hemodialysis in HD patients depend on the regular taking of medications such as phosphate binders, it is possible that alexithymia is linked to phosphatemia in HD patients in this manner [41]. Even though it was hypothesized that alexithymia would have a positive correlation with high phosphorus levels due to its effect on a lower degree of diet control, it is obvious that the relationship between alexithymia and the confounding factors is different [42]. Another possible mechanism that may explain the link between alexithymia and low pre-dialysis phosphorus levels is the low appetite of HD patients with alexithymia, which was concluded based on the relationship between alexithymia and eating disorders in patients with functional gastrointestinal disorders and eating disorders, such as anorexia and bulimia [43]. In studies conducted on patients with chronic kidney disease, the highest levels of alexithymia were observed in patients with bulimia as a comorbidity and who were likewise malnourished [44]. It is possible that low phosphorus levels in individuals with alexithymia are correlated with the lower appetite of HD patients, which is a factor that we did not control for in our study and which should be controlled for in further studies [45]. From all the above, we concluded that in our alexithymic patients, the appetite itself was reduced, they ate less, were malnourished, had eating disorders, and therefore, their phosphorus values were lower.

The prevalence of sleep disorders in our sample of HD patients was almost 69%, which was mostly in accordance with the results of previous studies. The prevalence of daytime sleepiness of around 21% in all HD patients that were involved was somewhat lower compared with other similar studies in which the prevalence of daytime sleepiness in HD patients reached up to 50% [46]. Given that our study used a subjective method of estimating daytime sleepiness in the form of the ESS, it is important to note that prior studies found no significant differences between subjective and objective methods of estimating daytime sleepiness [46]. The result of our study regarding the negative correlation between interdialytic weight gain and daytime sleepiness was surprising because this factor was correlated with obstructive sleep apnea and volume overload in previous studies [47]. We assumed that hypovolemia is linked to more pronounced daytime sleepiness due to blood pressure levels; therefore, we believe that a more adequate blood pressure measurement, such as continuous arterial pressure measurement, should be used in future studies. Additional research in this group of subjects is required since the combination of hypovolemia and excessive daytime sleepiness may influence HD patients’ cognitive function while doing everyday activities, taking medication, and using higher cognitive skills involving learning and memory [46].

Significantly higher levels of daytime sleepiness in the group of HD patients with alexithymia compared with HD patients without alexithymia represented a unique discovery, even when we consider studies that examined the relationship between alexithymia and daytime sleepiness in samples of the general population. In his study conducted on a sample of workers in Japan in 1997, Fukunishi showed that there existed a correlation between alexithymia and excessive daytime sleepiness [48]. Polysomnographic studies that resulted in findings of longer stage 1 NREM sleep and shorter duration of slow-wave NREM sleep in persons with alexithymia in the general population also showed higher levels of daytime sleepiness during the next day [49]. Authors of this study state that it is possible that the shorter duration of slow-wave sleep stages in individuals with alexithymia results in higher levels of fatigue and daytime sleepiness, which are the result of inadequate sleep in such individuals [49]. Our model also represents a unique and new finding, in which alexithymia, after controlling for factors that were connected with excessive daytime sleepiness in previous studies, such as age, gender, hemoglobin levels, sleep quality, depression, and interdialytic weight gain, was an independent predictive factor of excessive daytime sleepiness such that for every point on the TAS-26 questionnaire, the HD patient had a 1.05 times higher chance of excessive daytime sleepiness.

According to these results, alexithymia was an increasingly important factor for HD patients since its connection with daytime sleepiness can make daytime functioning in terms of self-care, safe movement, and cognitive functions of learning and memorizing more difficult, as excessive daytime sleepiness has a negative effect thereon [50].

## 5. Limitations and Lack of Studies

Our research was conducted in a cross-sectional design; therefore, we cannot claim that the observed variables were causally related, and thus, these relationships need to be examined in prospective design studies. The study’s disadvantage was that we did not measure pain as a factor that should be controlled in a clinical sample like this to influence alexithymia levels and because mostly self-assessment and assessment instruments were used to measure sleep quality and alexithymia without objective methods, such as polysomnography. The study was limited to subjects who met the study strength (1-β) of 0.8 for groups of HD patients based on alexithymia levels.

## Figures and Tables

**Table 1:** Values of the Toronto Alexithymia Scale 26.

	Median (Interquartile Range)	Minimum–Maximum
Difficulty describing feelings	15 (11–18)	5–34
Difficulty identifying feelings	28 (22–38)	7–58
Externally oriented thinking	12 (10–16)	3–32
Reduced daydreaming	15 (12–18.75)	8–22
Total Toronto Alexithymia Scale 26	68 (62–77)	27–114

**Table 2:** Alexithymia based on the patients’ ages.

	Median (Interquartile Range) According to TAS 26		Difference ^%^	95% Confidence Interval	*p* *
	Up to 62 points	Above 63 points			
Age of participants	54.5 (46–61)	73 (66–80)	−2	−7 to 2	0.318

* Mann–Whitney U test; ^%^ Hodges–Lehmann estimator.

**Table 3:** Levels of alexithymia based on the patients’ sex.

	Arithmetic Mean (Standard Deviation)		Difference ^%^	95% Confidence Interval	*p* *
	Men	Women			
Levels of alexithymia	69.38 (14.87)	68.04 (15.41)	1.33	−3.59 to 6.26	0.594

* Independent *t*-test; ^%^ Levene’s test for equality of variances.

**Table 4:** Evaluation of total sleep quality scale, sleep latency, and duration of sleep (in the number of points) of HD patients (PSQI).

	Median (Interquartile Range)	Minimum–Maximum
Total points on the sleep scale (PSQI)	6 (4–10)	0–21
Sleep latency	1 (1–2)	0–3
Duration of a sleep	1 (0–2)	0–3

PSQI—Pittsburgh Sleep Quality Index.

**Table 5:** Distribution of participants according to daytime sleepiness.

	Number (%) of Participants
Up to 10	124 (72.9)
11 or more (high daytime sleepiness)	35 (20.9)
Total	159 (93.5)

**Table 6:** Differences in interdialytic weight gain and blood pressure in comparison with daytime sleepiness (Epworth).

	Median (Interquartile Range) According to the Epworth Scale		Difference ^%^	95% Confidence Interval	*p* *
	Up to 10	Up to 10			
Interdialytic weight gain	0.0149 (0.0099–0.0196)	0.0124 (0.0099–0.0158)	−0.003	−0.005 to 0	0.05
Systolic blood pressure (mmHg)	137 (121–146)	137 (127–146)	3	−5 to 10	0.49
Diastolic blood pressure (mmHg)	78 (70–81)	77 (73–81)	0	−3.3 to 3.3	0.92

* Mann–Whitney U test; ^%^ Hodges–Lehmann estimator.

**Table 7:** Differences in biochemical indicators according to sleep quality (PSQI).

	Median (Interquartile Range) According to PSQI ^§^		Difference ^%^	95% Confidence Interval	*p* *
	Good Sleepers	Bad Sleepers			
CRP	2.8 (1–5.8)	5.6 (2.2–10.6)	1.6	0.18 to 3.76	0.02
Leukocytes	6.3 (4.85–7.3)	6.5 (5.2–8.33)	0.6	−0.1 to 1.3	0.11
Depression (HDRS)	4 (3–6.5)	9 (5–14)	−5	−7 to −2	0.00

* Mann–Whitney U test; ^%^ Hodges–Lehmann estimator; ^§^ PSQI—Pittsburgh Sleep Quality Index.

**Table 8:** Differences in interdialytic weight gain and pre-hemodialysis biochemical indicators.

	Median (Interquartile Range) Based on Alexithymia		Difference ^%^	95% Confidence Interval	*p* *
	Without Alexithymia (*n* = 45)	With Alexithymia (*n* = 109)			
Age (years)	66.5 (53–72)	67 (59–75)	2	−2 to 7	0.32
Interdialytic weight gain	0.0128 (0.009–0.0185)	0.0146 (0.01–0.0191)	0.001	−0.002 to 0.004	0.39
CRP	5.2 (1.5–10.8)	4.05 (1.8–9.8)	−0.29	−2.1 to 1.1	0.62
Leukocytes	5.9 (4.1–7.4)	6.5 (5.4–8.2)	0.9	0.2 to 1.6	0.02
Pre-HD phosphorus	1.71 (1.38–2.09)	1.45 (1.13–1.77)	−0.27	−0.44 to −0.08	0.005
Subjective sleep quality (PSQI ^§^)	1 (0.75–1)	1 (1–2)	0	0 to 0	0.10
Sleep latency	1 (1–2)	1 (1–2)	0	0 to 0	0.437
Duration of a sleep	1 (0–2)	1 (0–2)	0	0 to 1	0.316
Epworth Sleepiness Scale	4 (3–6)	8 (4–11)	3	1 to 4	0.001
Depression (HDRS ^$^)	5 (3–10)	8 (4–15)	−3	−6 to −1	0.01

* Mann–Whitney U test; ^%^ Hodges–Lehmann estimator; ^§^ PSQI—Pittsburgh Sleep Quality Index; ^$^ Hamilton Depression Rating Scale.

**Table 9:** Functional correlation of phosphorus with alexithymia (linear regression analysis).

	Bivariate Linear Regression			
	ß *	95% CI ^%^ ß	*p*	R^2^
Pre-HD phosphorus				
Alexithymia	−0.01	−0.01 to −0.004	0.001	0.079

* ß—regression coefficient; ^%^ CI—confidence interval.

**Table 10:** Prediction of the probability of daytime sleepiness in participants (Epworth Sleepiness Scale) (bivariate and multivariate regression analysis).

Predictive Factors	ß *	Standard Error	Wald	Odds Ratio (OR)	95% Confidence Interval	*p*
Bivariate Regression						
Age	0.001	0.015	0.01	1.01	0.97 to 1.03	0.94
Gender	0.286	0.401	0.509	1.33	0.60 to 2.92	0.48
Interdialytic weight gain	−65.74	29.75	4.82	0.00	0.00 to 0.001	0.03
Systolic blood pressure	0.01	0.011	0.42	1.01	0.98 to 1.03	0.52
Diastolic blood pressure	−0.002	0.023	0.01	0.99	0.95 to 1.04	0.94
Hemoglobin	−0.005	0.015	0.10	0.99	0.96 to 1.02	0.75
Alexithymia (scale)	0.49	0.014	11.6	1.05	1.02 to 1.08	0.001
Depression (HDRS ^§^)	0.02	0.034	0.002	1.002	0.93 to 1.07	0.96
Sleep quality (poor) (PSQI ^%^)	0.03	0.043	0.59	1.03	0.95 to 1.12	0.44
Sleep latency	0.28	0.225	1.530	1.321	0.85 to 2.05	0.21
Duration of a sleep	−0.43	0.22	3.87	0.65	0.42 to 0.99	0.049
Multivariate Regression (*Stepwise* Method)						
Alexithymia	0.049	0.018	7.336	1.051	1.014 to 1.089	0.007
Constant	−4.015	1.47	7.46			0.006

* ß—regression coefficient; ^%^ PSQI—Pittsburgh Sleep Quality Index; ^§^ Hamilton Depression Rating Scale.

## Data Availability

The data supporting the findings of this study are available from the corresponding author on request. The data are not publicly available due to their containing information that could compromise the privacy of participants.
